# Amphiphilic Fluorine-Containing Block Copolymers as Carriers for Hydrophobic PtTFPP for Dissolved Oxygen Sensing, Cell Respiration Monitoring and In Vivo Hypoxia Imaging with High Quantum Efficiency and Long Lifetime

**DOI:** 10.3390/s18113752

**Published:** 2018-11-02

**Authors:** Jiaze Li, Yuan Qiao, Tingting Pan, Ke Zhong, Jiaxing Wen, Shanshan Wu, Fengyu Su, Yanqing Tian

**Affiliations:** 1 School of Materials Science and Engineering, Harbin Institute of Technology, Nangang District, Harbin 150001, China; 11749240@mail.sustc.edu.cn; 2 Department of Materials Science and Engineering, Southern University of Science and Technology, Xili, Nanshan District, Shenzhen 518055, China; qiaoy@mail.sustc.edu.cn (Y.Q.); 11553010@mail.sustc.edu.cn (T.P.); 11612419@mail.sustc.edu.cn (K.Z.); 11510869@mail.sustc.edu.cn (J.W.); 2018090123@gdip.edu.cn (S.W.); 3 Light Chemical Technology College, Guangdong Industry Polytechnic, Haizhu District, Guangzhou 510300, China; 4 SUSTech Academy for Advanced Interdisciplinary Studies, Southern University of Science and Technology, Xili, Nanshan District, Shenzhen 518055, China

**Keywords:** dissolved oxygen sensors, fluoropolymers, micelles, cell respiration monitoring, tumor imaging

## Abstract

New amphiphilic star or multi-arm block copolymers with different structures were synthesized for enabling the use of hydrophobic oxygen probe of platinum (II)-tetrakis (pentafluorophenyl) porphyrin (PtTFPP) for bioanalysis. The amphiphilic star polymers were prepared through the Atom Transfer Radical Polymerization (ATRP) method by using hydrophilic 4-arm polyethylene glycol (4-arm-PEG) as an initiator. Among the five block copolymers, **P1** series (**P1a**, **P1b**, and **P1c**) and **P3** possess fluorine-containing moieties to improve the oxygen sensitivity with its excellent capacity to dissolve and carry oxygen. A polymer **P2** without fluorine units was also synthesized for comparison. The structure-property relationship was investigated. Under nitrogen atmosphere, high quantum efficiency of PtTFPP in fluorine-containing micelles could reach to 22% and long lifetime could reach to 76 μs. One kind of representative PtTFPP-containing micelles was used to detect the respiration of *Escherichia coli* (*E. coli*) JM109 and macrophage cell J774A.1 by a high throughput plate reader. In vivo hypoxic imaging of tumor-bearing mice was also achieved successfully. This study demonstrated that using well-designed fluoropolymers to load PtTFPP could achieve high oxygen sensing properties, and long lifetime, showing the great capability for further in vivo sensing and imaging.

## 1. Introduction

Dissolved oxygen (DO) is crucial to environment, industry, life technology, and human health, etc. Hypoxia [1] (≤0.5% O_2_, usually at tumor environments) is relevant to cancer, stroke [2], arteriosclerosis [3], Parkinsonism [4], Alzheimer’s disease [5], etc. It has been reported that oxygen played an important role when methyl group from DNA was removed by enzymes. Otherwise, hypermethylation could result in abnormal cell behavior and rapid growth of tumors [6]. At the gene level, the hypoxia-inducible factor-1α (HIF-1α) maintained the in vivo oxygen homeostasis [7,8]. On the other hand, hypoxia can also influence clinical surgery such as radiotherapy, because low-level oxygen condition will decrease the radiation efficiency [9]. Until now, DO sensing and imaging have remained to be a challenge in biology, medicine, the environment and the food industry. There are several ways to detect dissolved oxygen such as Clark electrodes [10], Winkler titration [11] and optical sensors [12]. The first two methods have been developed maturely, but there are still limitations. Clark electrode can only measure the relative oxygen content of air and samples [13], while Winkler titration is unsuitable for real-time continuous oxygen detection [14], and both approaches consume oxygen during detection. Phosphorescence based optical oxygen analysis makes up for the shortfalls of the first two methods with the following characters: (1) noninvasive and reversible sensing; (2) without oxygen consumption; (3) high sensitivity to oxygen with fast responses; and (4) capable for single cell level sensing. These properties gave huge potentials for phosphorescence oxygen sensing in biological application [15,16,17].

Metalloporphyrin derivatives are typical oxygen sensors possessing excellent optical stability and significant oxygen quenching properties. For instance, platinum (II)-tetrakis (pentafluorophenyl) porphyrin (PtTFPP) as one of the best oxygen probes has a long lifetime and super photo bleaching resistance [18]. However, PtTFPP is extremely hydrophobic, resulting in its unsuitableness for in vitro and further in vivo dissolved oxygen sensing. In order to enable the application of PtTFPP and its derivatives for biostudies, scientists have explored a few approaches along this direction, including the generation of nanoparticles with the assistance of conjugated polymers [19,20], the conjugation of PtTFPP-derivatives into the main-chain of conjugated polymers [21,22,23], and the encapsulation of the porphyrin probe into the micelles formed from amphiphilic block or graft copolymers [24], etc. We have been using the micelle approach to transfer PtTFPP into aqueous solution for oxygen sensing by using linear block copolymer and graft copolymers [25,26,27]. However, there is no systematical investigation of structure-property relationships.

Considering the abundance of polymer structures, herein we extended this study to multi-arm block copolymers. A modified 4-arm polyethylene glycol (4-Arm-PEG) as a hydrophilic chain was used as an initiator to polymerize three kinds of monomers to achieve five kinds of block copolymers with different structures. We chose two kinds of fluorine-containing monomers with different fluorine-units for hydrophobic chains and one fluorine-free monomer (styrene) for comparison. Thus structure-properties can be evaluated. The use of multi-arm block copolymers is due to their good stability, high drug loading ratios and long in vivo circulation time [28,29]. On the other hand, fluorine is super hydrophobic. It has been reported that micelles formed from fluorine-containing amphiphilic polymers exhibited high stability and nontoxicity which can be used as protein drug carriers [30]. Based on the former research on perfluorocarbon oxyglobin, fluorine has greater capacity to dissolve, carry and permeate oxygen [31]. Besides, the presence of halide substituents could increase the photostability of metalloporphyrins [32]. Therefore, these fluorine-containing amphiphilic block copolymers are expected to have excellent compatibility with the fluorine-containing PtTFPP to achieve high quantum efficiencies of the PtTFPP in aqueous solution as well as high oxygen sensitivity. After evaluating the structure-properties relationship, representative micelles will be used for cell respiration monitoring and in vivo hypoxia imaging.

## 2. Materials and Methods

### 2.1. Materials and Reagents

PtTFPP was purchased from Frontier Scientific (Logan, UT, USA). Hexafluorobutyl methacrylate (HFMA) stabilized with hydroquinone monomethyl ether (MEHQ) and α-bromoisobutyryl bromide were acquired from TCI (Tokyo, Japan). Styrene stabilized with 4-tert-buytlcatechol (TBC) and trifluoroethyl methacrylate (TFEMA) stabilized with MEHQ were obtained from Aladdin (Shanghai, China). The 4-arm-PEG-OH, 4-(2-hydroxyethyl)-1-piperazineethanesulfonic acid (HEPES), 1,1,4,7,7-pentamethyl-diethylenetriamin (PMDETA), and CuBr were purchased from Creative PEGWorks (Chapel Hill, NC, USA), Sigma-Aldrich (St. Louis, MO, USA), Energy Chemical (Shanghai, China), and J & K Scientific Ltd. (Shanghai, China), respectively. All the monomers used for polymerization were purified by passing through a basic Al_2_O_3_ column to remove the stabilizer before used. The monocyte macrophage J774A.1 cell line was provided by GuangZhou Jennio Biotech Co., Ltd. (Guangzhou, China). The human breast adenocarcinoma cell line MCF-7 was obtained from Shanghai Zhong Qiao Xin Zhou Biotechnology Co., Ltd (Shanghai, China).

### 2.2. Instruments

Water 1515 gel permeation chromatography (GPC) coupled with a refractive index (RI) detector (Waters, Milford, MA, USA) and nuclear magnetic resonance spectroscopy (NMR) (Bruker Avance III 400M, Karlsruhe, Germany) were used to determine relative molecular weights of polymers. Dynamic light scattering (DLS) (Malvern Nano ZS, Malvern, WR, UK) was used to measure the diameters of micelles. Fluorescence Spectrofluorophotometer (PerkinElmer LS 55, Shelton, CT, USA) was applied to measure photo-luminescence intensities. A gas manipulator (Alicat Scientific Instrument, Tucson, AZ, USA, measuring error: <1%) was used for controlling the exact gas percentage during phosphorescence measurements. Transient Absorption Spectrometer (FLS 980, Edinburch Instruments Ltd., Livingston, UK) was used to detect phosphorescence lifetimes. A Plate Reader (BioTek Cytation 3, Winooski, VT, USA) was used for toxicity test and respiration measurements of *Escherichia coli* (*E. coli*) and cells.

### 2.3. Synthesis of Macroinitiator 4-Arm-PEG-Br and Amphiphilic Star Polymers

**Scheme 1:**
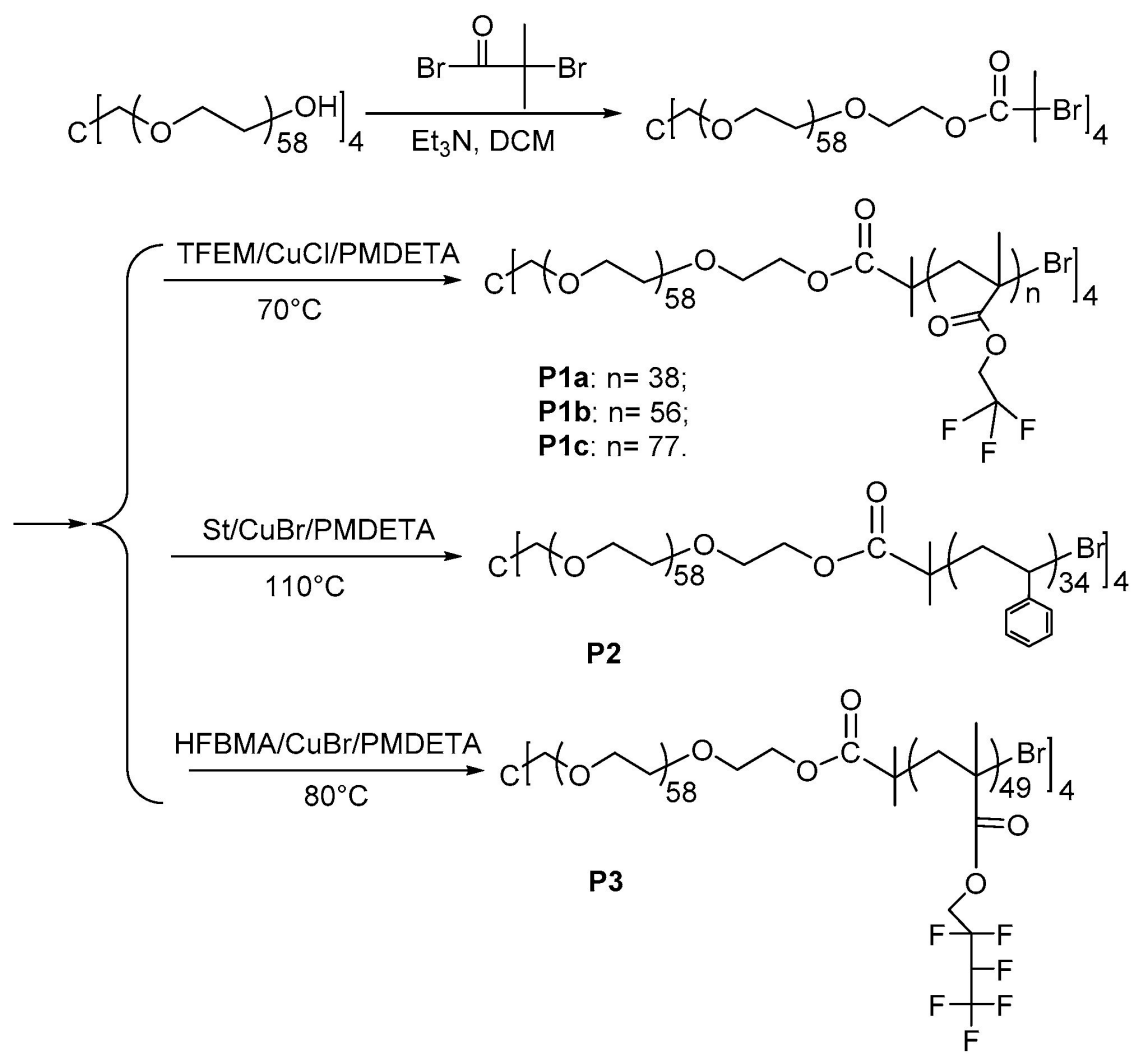
Synthesis of 4-arm-PEG- Br macroinitiator and its star block copolymers.

Macroinitiator 4-arm-PEG-Br (Scheme 1): 5.0 g (0.5 mmol) of 4-Arm-PEG-OH and 0.7 mL (5 mmol) of Et_3_N were added into 100 mL of ultra-dry dichloromethane (DCM). A total of 0.6 mL (5 mmol) of α-Bromoisobutyryl bromide was dissolved in 25 mL of anhydrous DCM and slowly dropped into the mixture solution in an ice bath under stirring. After 24 h, the mixed solution was precipitated in ice cooled ether to get the macroinitiator. The crude product was dissolved in water (pH = 9) and extracted by dry DCM to remove the triethylamine hydrochloride thoroughly. Yield: 79%. ^1^H NMR (400 MHz, CDCl_3,_ δ (ppm)): 4.33 (t, 2 H, -C**H**_2_OCO-), 3.64 (m, 230 H, -OC**H**_2_C**H**_2_O-), 1.94 (s, 6 H, -CC**H**_3_-). M_n(NMR)_ = 10,940, M_n(GPC)_ = 3960, M_w(GPC)_/M_n(GPC)_ = 1.1.

**P1a**: A total of 0.266 g (0.025 mmol) of the 4-arm-PEG-Br and 10 mg (0.1 mmol) of CuCl were added into a Schlenk tube, and the Schlenk tube was vacuumed for 15 min. A solution of 0.58 mL (5 mmol) of trifluoroethyl methacrylate and 20 μL (0.1 mmol) of PMDETA in 3 mL of anisole was slowly injected into the Schlenk tube. After three times freeze-thaw procedure to remove residual oxygen, the Schlenk tube was placed in a 70 °C oil bath for 24 h. After the polymeric solution was passed through neutral Al_2_O_3_ column to remove copper, the polymer was obtained by precipitating in excess cold n-hexane to get **P1a** with a yield of 50%. ^1^H NMR (400 MHz, CDCl_3_, δ (ppm)): 4.34 (s, 76 H, -OC**H**_2_CF_3_-); 3.64 (m, 230 H, -OC**H**_2_C**H**_2_O-). M_n(NMR)_ = 33,790, M_n(GPC)_ = 5340, M_w(GPC)_/M_n(GPC)_ = 1.3.

**P1b** was obtained by the same method as **P1a**, except the feed ratio of monomer to the macroinitiator was 400:1. Yield: 44%. ^1^H NMR (400 MHz, CDCl_3,_ δ (ppm)): 4.34 (s, 112 H, -OC**H**_2_CF_3_-); 3.64 (m, 230 H, -OC**H**_2_C**H**_2_O-). M_n(NMR)_ = 45,880, M_n(GPC)_ =7650, M_w(GPC)_/M_n(GPC)_ = 1.2.

**P1c** was attained by the same way as **P1a**, except the feed ratio of monomers to the macroinitiator was 600:1. Yield: 40%. ^1^H NMR (400 MHz, CDCl_3,_ δ (ppm)): 4.34 (s, 154 H, -OC**H**_2_CF_3_-); 3.64 (m, 230 H, -OC**H**_2_C**H**_2_O-). M_n(NMR)_ = 62,680, M_n(GPC)_ =10,710, M_w(GPC)_/M_n(GPC)_ = 1.3.

**P2** was synthesized through the same method as **P1a**, however using styrene as the monomer and the reaction temperature was set at 110 °C. Yield: 26%. ^1^H NMR (400 MHz, CD_2_Cl_2,_ δ (ppm)): 6.46–7.08 (m, 171 H, Ar**H**); 3.58 (m, 230 H, -OC**H**_2_C**H**_2_O-). M_n(NMR)_ = 24,780, M_n(GPC)_ = 6280, M_w(GPC)_/M_n(GPC)_ = 1.2.

**P3** was acquired by the same method as **P1a**, however using hexafluorobutyl methacrylate as the monomer and the reaction temperature was set at 80 °C. Yield: 48%. ^1^H NMR (400 MHz, CDCl_3,_ δ (ppm)): 4.85 (m, 49 H, -C**H**F-); 4.35 (s, 96 H, -OC**H**_2_-); 3.64 (m, 230 H, -OC**H**_2_C**H**_2_O-). M_n (NMR)_ = 59,220, M_n(GPC)_ = 8370, M_w(GPC)_/M_n(GPC)_ = 1.5.

### 2.4. Preparation of Micelles with PtTFPP

A total of 30 mg of each polymer in 1.2 mL of tetrahydrofuran (THF) and 2 mg of PtTFPP in 200 µL of THF were mixed then added slowly into 6 mL of deionized water under fast stirring. The solutions were dialyzed against water for 2 days. Then, the solutions were passed through 0.45 μm filter membranes and stored in a brown bottle at 4 °C for further use. For biological application, the micelles were dialyzed again against HEPES solution for 2 days. The micelles formed from **P1a**, **P1b**, **P1c**, **P2** and **P3** were named as **M1a**, **M1b**, **M1c**, **M2** and **M3**, respectively. The concentration of PtTFPP packed in micelles was detected by UV-Vis Absorption Spectra.

### 2.5. Critical Micelle Concentrations Determination of Block Polymers

As there is no luminescent group in these amphiphilic polymers, we used pyrene approach to determine the critical micelle concentrations (CMC) of the block copolymers. The fluorescence spectrum of pyrene has five peaks [33,34]. The intensity ratio of the first peak (373 nm) to the third peak (384 nm) will decrease with the decline of solution polarity. Thus, CMC was measured by mixing pyrene in different concentrations of polymer solutions, and the I_373_/I_384_ value will decrease to a constant when and the pyrene transferred from water to micelles, which means the formation of pyrene-containing micelles.

### 2.6. Quantum Efficiency Determination

Quantum efficiency measurement was carried out under nitrogen atmosphere. As is reported in former literature, quantum efficiency of PtTFPP in DCM is 0.088 [35]. Setting this value as a reference, we can calculate PtTFPP-containing micelles’ quantum efficiencies by using the emission intensity and absorbance of PtTFPP in micelles and in DCM, respectively (Equation (1)).

(1) $$\eta_{s}=\eta_{r}\left(\frac{A_{r}}{A_{s}}\right)\left(\frac{I_{s}}{I_{r}}\right)\left(\frac{n_{s}^{2}}{n_{r}^{2}}\right).\;$$

In the Equation (1) [36], *η_r_* is the quantum efficiency of PtTFPP in DCM; *n_r_* is the refractive index of DCM (*n_r_* = 1.424); *n_s_* is the refractive index of water (*n_s_* = 1.333); *A_r_* and *A_s_* indicate the absorbance of reference and samples respectively; *I_r_* and *I_s_* indicate the phosphorescence intensities of reference and samples, respectively.

### 2.7. Response to Oxygen

Due to the special oxygen quenching property of PtTFPP, higher oxygen concentration results in lower emission intensity. A total of 3 mL of Micelle solution was placed in a quartz cell, oxygen concentration was adjusted by bubbling mixed gases of oxygen and nitrogen with various ratios into the micelle solutions. Emission spectra were measured under an excitation wavelength of 405 nm.

### 2.8. Response Time Measurement

Similar to the oxygen titration, we detected the dynamic relationship between phosphorescence intensity and time through phosphorescence spectra (excitation: 405 nm; emission: 650 nm). We defined the response time as the time corresponding to the intensity that 95% of the difference between maximum intensity and minimum intensity when the atmosphere switched between 100% oxygen and 100% nitrogen.

### 2.9. Phosphorescence Lifetime Determination

A Transient Absorption Spectrometer was used to measure the phosphorescence decay time of PtTFPP-containing micelles under air and nitrogen respectively.

### 2.10. E. coli Culture

*E. coli* JM109 was cultivated in Luria-Bertani (LB) medium at 37 °C under shaking. Through measuring the *E. coli* optical density (OD) value (from 0.1 to 1) at 600 nm by UV-Vis Absorption Spectrometer, the density of *E. coli* was obtained. OD_600_ of 1 equals to 5 × 10^8^ cfu/mL. Appropriate densities were diluted with LB medium.

During toxicity measurement, several densities of *E. coli* were added into a 96-well plate. The micelles added into each well contained 10 μg/mL of PtTFPP. The wells without sensors were set as the control group. The experiment was executed through a plate reader at 37 °C with an oil seal. In another group of parallel experiments, 4 × 10^7^ cfu/mL of *E. coli* was added to another 96-well plate, while different concentrations of the PtTFPP-containing micelles were added.

In the respiration detection, we also used a plate reader to collect the changes in phosphorescence intensity over time among different densities of *E. coli*. At the early stage of the experiment, the emission intensity will decrease due to the temperature equilibrating issue or temperature effect [26,27], so we stabilized the samples for 20 min before the detection.

### 2.11. Cell Culture

J774A.1 cells were cultured in a mixture medium containing 89.1% of Dulbecco’s modified eagle medium (DMEM) medium, 9.9% of fetal bovine serum (FBS) and 1% of penicillin-streptomycin. After being cultivated at 37 °C in 5% of CO_2_ incubator, cells were vaccinated on 96-well plates for toxicity and respiration experiments.

MCF-7 cells were cultured in the same medium as J774A.1. After being cultivated at 37 °C in 5% of CO_2_ incubator, MCF-7 cells were used for tumor formation in the animal experiment.

### 2.12. Mice Culture and In Vivo Imaging

Female Balb/c nude mice were purchased from Beijing Vital River Laboratory Animal Technology Co., Ltd. and fed in a Specific Pathogen Free (SPF) animal facility. A total of 100 μL of MCF-7 cells (1 × 10^7^ cells/mL) were injected into the left hind leg of each Balb/c nude mouse. Two weeks after MCF-7 cells injection, tumor imaging was carried on through an In Vivo Imaging System. Before imaging, the PtTFPP-containing micelles were injected into the tumor and subcutis of mice, respectively.

The animal experiment followed the Guidelines for the Care and Use of Research Animals established by the Southern University of Science and Technology Laboratory Animal Center (SUSTC-JY2018078).

## 3. Results and Discussion

### 3.1. Design and Synthesis of Block Copolymers

**Figure 1:**
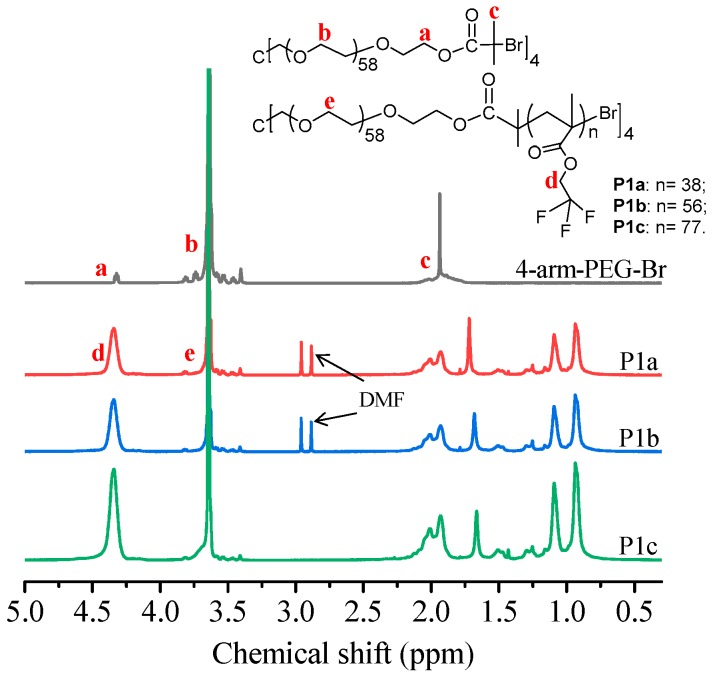
^1^H NMR spectra of 4-arm-PEG-Br, **P1a**, **P1b**, and **P1c**.

A few kinds of 4-arm amphiphilic block copolymers were synthesized (Scheme 1). These amphiphilic block copolymers can form micelles for encapsulating hydrophobic PtTFPP into their micelles to enable the application of PtTFPP in aqueous conditions. Some studies revealed that the micelles formed from multi-arm block copolymers may exhibit more morphologies and assemblies than linear polymers [37,38] as well as better stability and longer in vivo cycle time [28,29]. Herein we explore whether these amphiphilic 4-arm block copolymers can be used for systemically studying structure-property relationships in the field of oxygen sensing and for cell metabolism related research. For understanding structure-property relationships, five block copolymers were synthesized. **P1a**, **P1b**, **P1c**, and **P3** possess fluorine moieties. The side-chain of **P3** possesses more fluorine units than those of **P1** series. **P2** does not contain fluorine moieties. Polymers with fluorine units are expected to have better compatibility with the hydrophobic fluorine-containing-PtTFPP to achieve high quantum efficiency and also for excellent sensitivity. Hydrophobic-hydrophilic ratios were also tuned to see whether these ratios have influences on photo-physical properties by using **P1a**, **P1b**, and **P1c** as three typical polymers. The block copolymers were synthesized by using ATRP—a kind of controlled living polymerization [39,40,41]. Through the tuning monomers and/or monomer/macroinitiator molar ratios, five different polymers were synthesized successfully. The polymers were characterized by using NMR and GPC. Their typical ^1^H NMR spectra and ^19^F NMR spectra were given in Figure 1, Supplementary Figures S1 and S2. Detailed data of these polymers were given in Table 1.

### 3.2. Critical Micelle Concentrations Determination

The CMC is an important factor for micelle formation and stability. CMCs of these polymers were determined by using the traditional Pyrene-encapsulation approach that we mentioned in the experiments section. Through the determination of pyrene’s aggregations in micelles by observing its excimer formation and ratios, CMCs were determined to be 5.7 to 10.5 µg/mL (Table 1), slightly affected by polymer structures. The ranges of CMC were comparable to some reported linear block copolymers [42], showing that these multi-arm bock copolymers’ micelles exhibit similar stability with other common linear block copolymers. Structure influences on the CMCs were observed. Longer hydrophobic segments induced slightly larger CMC as compared within the **P1** series. Most likely, the length of PEG may uneasily stabilize the longer hydrophobic segments in **P1** series, to result in higher CMC in higher molecular weight polymers.

### 3.3. Micelle Preparation and Their Photophysical Properties

**Figure 2:**
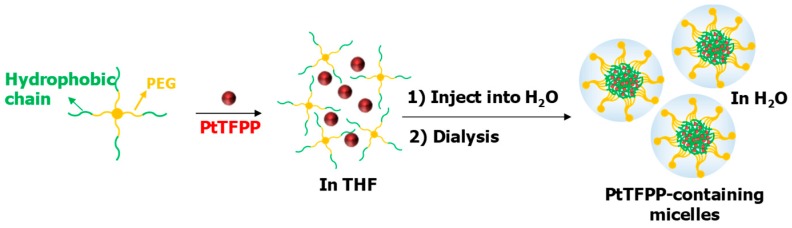
The preparation process of micelles.

The preparation of micelles was illustrated in Figure 2. The hydrophobic segments of the polymers formed micellar cores and the hydrophilic PEG segments formed hydrophilic shells to encapsulate the hydrophobic PtTFPP in the micellar cores. This approach enabled the application of PtTFPP in aqueous solutions.

**Figure 3:**
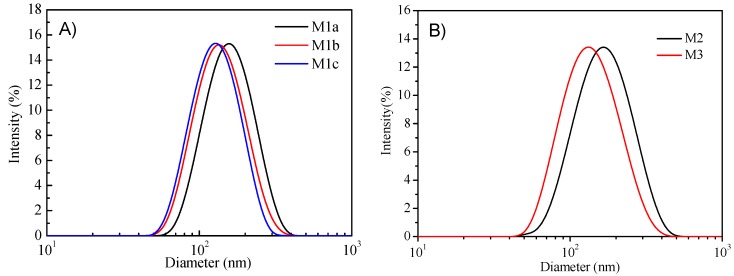
Dynamic light scattering (DLS) results of the PtTFPP-containing micelles: **M1b**, **M1c** (**A**) and **M2**, **M3** (**B**).

The DLS was used to measure micelles diameters (Figure 3). Detailed data were given in Table 2. It was found that the micelles’ diameters were in the range of 122 to 163 nm. **P1c** has the lowest encapsulation efficiency for PtTFPP, although it has the highest molecular weights among the **P1** series polymers. This might be due to the poor solubility of **P1c** in water with the longest hydrophobic chain. Additionally, the encapsulation efficiency for PtTFPP for all the three **P1** series polymers is smaller than the polymers with polystyrene chains (**P2**) and longer alkyl chains with more fluorine units (**P3**), showing the polymer structures’ influence on sensing probes loading.

Quantum efficiencies of the PtTFPP molecules in these micelles could be achieved as high as 22.3% by using the fluorine-containing polymers **P1** series. Among the five polymers, **P2**’s micelles without fluorine moieties possess the lowest quantum efficiency of 11.8%. This comparison indicates the advantage of the use of the fluorine-containing polymers for achieving high quantum efficiency of PtTFPP in aqueous solutions. This result was also supported by the long lifetime of the PtTFPP in fluorine-containing micelles (70–76 μs for **P1a**, **P1b**, **P1c**, and **P3** under nitrogen, Table 2 and Figure S3), because of the high hydrophobicity and compatibility of the fluorine moieties in the hydrophobic side-chains with the fluorine-containing PtTFPP. Previous studies showed that the phosphorescence lifetime of a well-designed nano-metal organic framework (MOF) probe which contained Pt(II)-porphyrin ligand as an O_2_ sensitive part and Rhodamine B isothiocyanate ligand as an O_2_-insensitive part was 28.1 μs under deoxygenated atmosphere [43]; a conjugated Pdots with fluorescence resonance energy transfer (FRET) exhibited 33.7 μs phosphorescence lifetime under nitrogen [21]; and an intracellular nanoparticle probe composed of cationic polymer as shell and PtTFPP as core possessed 70 μs hypoxia phosphorescence lifetime attributed to the excellent loading property of the purchased polymer [44]. Lehner et al. reported an oxygen sensing device which embedded PtTFPP in a polymer matrix to improve the lifetime of PtTFPP to 90 µs, however, it is bulk material instead of nanoparticle [45]. Therefore, the lifetime in the range of 70–76 µs reported here is among the longest ones in the known literature, showing the advantage of the use of fluorine-containing multi-arm block copolymers. On the other hand, previously we reported that PtTFPP in micelles could achieve high quantum efficiency of 20–23%, however through FRET enhancement [27,30].

It is worth noting here that metalloporphyrin’s hydrophobicity results in their difficulty for application in water. Previous studies showed that water-soluble platinum porphyrins’ quantum efficiency was less than 1% in water [46]. Dendrimer’s approach was utilized to prevent the interaction of the platinum porphyrin units with water to achieve high quantum efficiency of 7.3% [47], however, dendrimers require long synthesis steps and a further purification process. Ruslan et al. reported the use of conjugated polymer nanoparticles to achieve high quantum yield of 18% of platinum porphyrin-derivatives [23].

Therefore, through the above discussion, this study showed the advantage of using fluorine-derived multi-arm amphiphilic block copolymers for transferring hydrophobic PtTFPP into aqueous system for oxygen sensing.

### 3.4. Oxygen Sensing Properties

**Figure 4:**
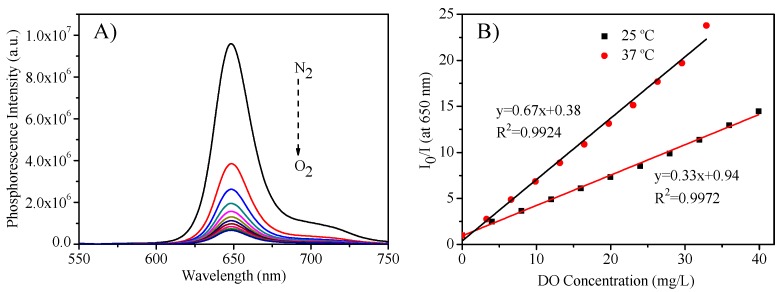
(**A**): Oxygen responses of **M3** at 25 °C; (**B**): Stern-Volmer plot of **M3** to oxygen at 25 °C and 37 °C. *I*_0_ is PtTFPP maximum phosphorescence intensity at 650 nm under hypoxia condition; *I* is the intensity at 650 nm under various dissolved oxygen (DO) concentrations.

The micelles’ response to oxygen was measured by adjusting the DO concentrations through the bubbling mixed oxygen and nitrogen into the micellar solutions. Figure 4A showed the representative oxygen responses by using the micelles formed from **P3** and PtTFPP (**M3**) at 25 °C. It was found that with the increase of oxygen concentration, the emission intensity decreased. Since most biological application was carried out at 37 °C, the sensing response was also measured at 37 °C, and a similar phenomenon was observed (Figure S4). The responses followed the linear Stern-Volmer equation (Equation (2) and Figure 4B), where *I*_0_ is PtTFPP’s maximum intensity at 650 nm under nitrogen; *I* is the intensity at 650 nm under various DO concentrations, *K_SV_* is the Stern-Volmer constant, [O_2_] is the dissolved oxygen concentration.

(2) $$\frac{I_{0}}{I}=1+K_{SV}[O_{2}].\;$$

Similar to other oxygen sensors [48], the sensitivity at 37 °C was higher than that at 25 °C, because of the more interaction activity of the oxygen molecules with the probe of PtTFPP at higher temperature. The sensitivity (*I*_0_/*I*_100_) using the intensity ratio under nitrogen and oxygen was given in Table 2 also. It can be found that the sensitivity of the micelles with fluorine-containing polymers is higher than that of **P2** without fluorine moieties, further exhibiting the suitableness and advantage of the fluorine-containing amphiphilic block copolymers as carriers for PtTFPP for sensing.

The lifetime of the PtTFPP in micelles was also evaluated briefly under air and hypoxia conditions, respectively. Figure S3E showed the representative changes of the **P3** micelles, the lifetime of **M3** was 18 μs under the air condition and 76 μs under the nitrogen condition (Table 2). Therefore, these PtTFPP-containing micelles formed from 4-arm fluoropolymers were suitable for long lifetime imaging which possesses high contrast, high sensitivity, no autofluorescence and no background interference [49].

**Figure 5:**
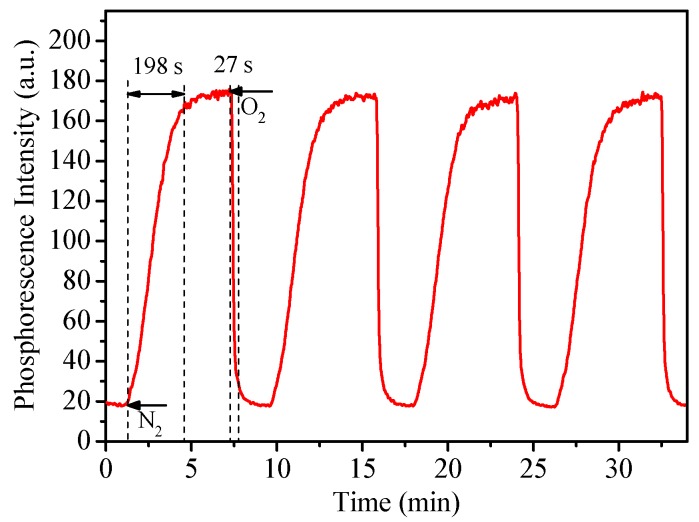
Dynamic phosphorescence intensity curve of **M3** when the atmosphere switches between 100% of oxygen and 100% of nitrogen.

Sensors’ response time was evaluated by alternating the oxygenated and deoxygenated conditions through the bubbling of nitrogen and oxygen into the solutions. Figure 5 showed the representative response time and reversibility test by using micelles **M3**. The response time from the deoxygenated condition to oxygenated condition at 95% equilibrium (t_95_) was 27 s, while the reverse one was 198 s (t_95-r_). The t_95_s for these micelles (27–34 s, Table 2) were generally in accordance with those of 24–34 s reported by using other micelles previously [26,27]. We also measured **M3**‘s response time with glucose oxidase (Figure S5). The result showed that when the activities of glucose oxidase (GOx) were 400, 100, 5, 2.5 and 1 U/mL, the times of that **M3** required to reach maximum intensity were 6 s, 20 s, 50 s, 90 s, 350 s, respectively. Results showed that when the enzyme concentration is high enough, the response of **M3** to oxygen consumption is very fast. The integration of the gas-equilibration and glucose-oxidation methods demonstrated that our sensor is sensitive to real-time oxygen concentration changes.

### 3.5. The Use of the Sensors for Monitoring Cellular Respiration

#### 3.5.1. Monitoring the Oxygen Consumption during the Growth of *E. coli* and the Cytotoxicity Evaluation

**Figure 6:**
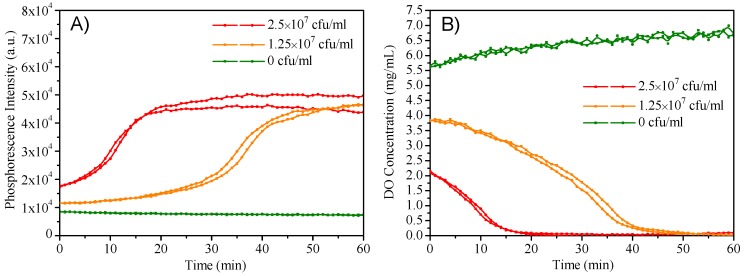
(**A**): Dynamic phosphorescence intensity curve of **M3** measured by plate reader to determine the *E. coli* respiration. (**B**): Extracellular oxygen consumption by *E. coli*.

**M3** was added into the LB cell culture medium with different densities of *E. coli* JM109 cells to monitor the oxygen consumption during the cell growth. For accomplishing the experiment faster, a thin layer of mineral oil was added on the top of the LB medium to eliminate the exchange of oxygen in the medium with air. The contrast experiment without the use of mineral oil was also performed and experimental data were given in Figure S6. Figure 6A showed the time-dependent emission intensity changes with the use of mineral oil, respectively. Higher cells induced earlier phosphorescence intensity changes and reached emission maximum within a shorter time; while the phosphorescence intensity without *E. coli* was not increased. Figure 6B showed the corresponding oxygen concentration changes. *E. coli* with a higher density consumed oxygen faster, showing the capacity of the micelles for cell respiration monitoring.

**Figure 7:**
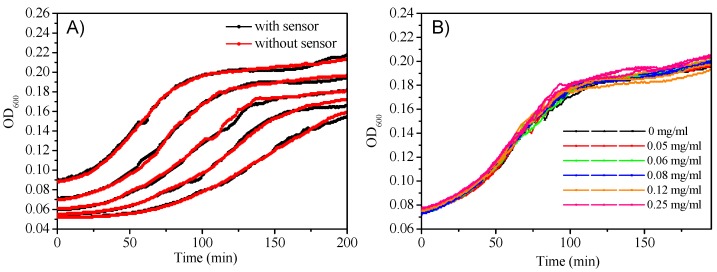
(**A**): Cell growth of *E. coli* with and without **M3** monitored by OD_600_; (**B**): Cell growth of *E. coli* with a consistent of cell density however different concentrations of **M3** monitored by OD_600_.

Micelles’ potential cytotoxicity to *E. coli* was evaluated by monitoring cell density changes with and without sensors. Figure 7A gave the cell growth curve with a consistent PtTFPP concentration (10 μg/mL) at different *E. coli* densities. Figure 7B plotted the *E. coli* growth curve with a defined *E. coli* density but using various sensors concentrations. No *E. coli* growth inhibition was observed, showing the nontoxicity of the sensor to *E. coli*.

#### 3.5.2. Monitoring Cell Respiration of J774A.1 Cells

**Figure 8:**
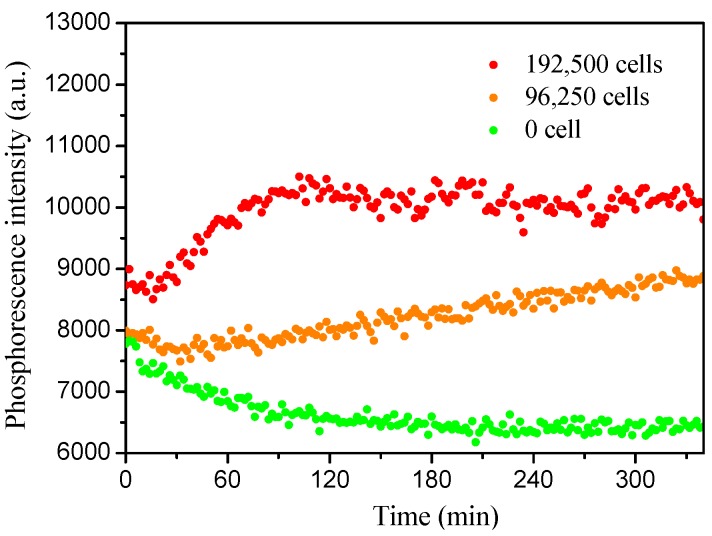
Cell respiration measurements of macrophages J774A.1 cells. Each well contained 10 μg/mL of PtTFPP in **M3** and was sealed with mineral oil. The phosphorescence intensity (excitation: 405 nm, emission: 650 nm) was measured by a plate reader at 37 °C.

**Figure 9:**
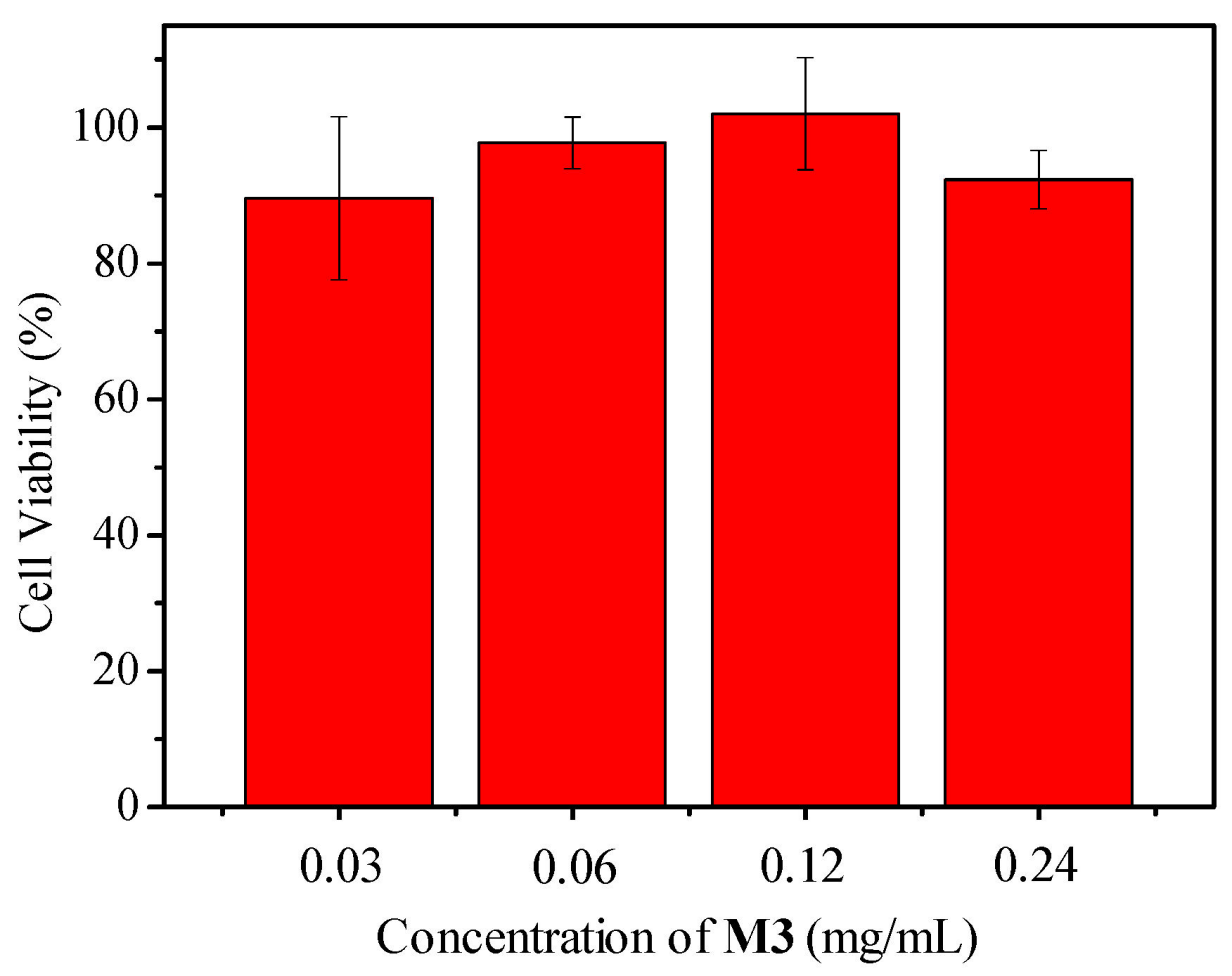
Viability of J774A.1 cells to different M3 concentrations (0.24 mg/mL of M3 contained 0.04 mg/mL of PtTFPP) after 24 h cell internalization measured by MTT assay. Initial cell concentration was 10,000 cells per well in a 96 well plate. Each measurement was repeated five times.

We also extended the sensor’s application to mammalian cells by using J774A.1 as a representative cell line. Figure 8 showed the time and cell concentration-dependent emission intensity changes. Similar to the phenomenon of *E. coli*, higher cell numbers induced faster oxygen consumption. Cell cytotoxicity was evaluated by using 3-(4,5-dimethyl-2-thiazolyl)-2,5-diphenyl-2-H-tetrazolium bromide (MTT) assay (Figure 9). No obvious cell cytotoxicity was observed.

### 3.6. In Vivo Hypoxia Imaging

**Figure 10:**
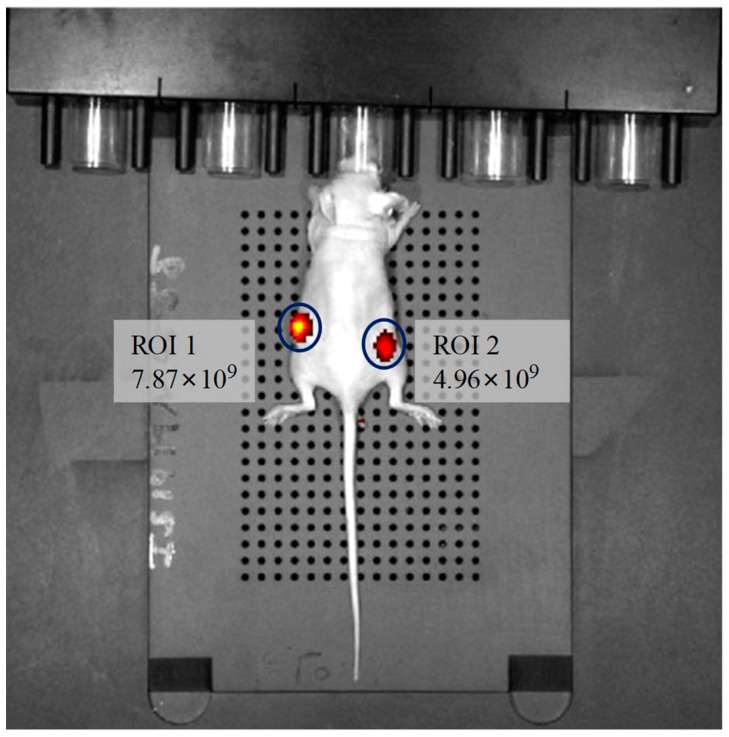
In vivo imaging of a tumor-bearing nude mouse after injection of **M3**, collocating with a bandpass filter (excitation: 430 ± 15 nm, emission: 640 ± 10 nm). ROI 1 was tumor and ROI 2 was normal skin.

Besides the use of the sensors for in vitro studies, we explored whether the sensors are suitable for in vivo hypoxia imaging. The mouse was intratumorally injected 50 μL (contained 0.3 mg/mL of PtTFPP and 1.9 mg/mL of **P3**) of **M3** (Figure S7 showed **M3** injection did not influence the change trend of mice’s weight within 7 days, so **M3** was nontoxic to tumor-bearing mice). After anesthesia, the phosphorescence image and intensity were gained by In Vivo Imaging System (Figure 10). Region of interest (ROI) 1 is the tumor region and ROI 2 is the normal region. It can be seen that the phosphorescence image at ROI 1 is much brighter. The phosphorescence intensity from ROI 1 is 1.6 times higher than that of ROI 2. This result demonstrated that the phosphorescence signal from oxygen sensors at the hypoxia site is stronger than the normal sites, indicating the sensors can be used for tumor diagnosis and imaging because the oxygen concentration at tumor sites are often under the hypoxia situation [50]. Moreover, the phosphorescence imaging lasted for at least 10 min without obvious decay of the phosphorescence signal after **M3** injection (Figure S8), indicating that the PtTFPP possessed excellent in vivo stability and strong phosphorescence signal after being encapsulated by **P3**. Thus, the sensors reported here might be capable for in vivo tumor imaging. It is expected that the further use of the sensors can be extended to measure the intravascular oxygen contents, which will be more instructive to cancer prevention and cancer clinical analysis.

## 4. Conclusions

Five amphiphilic multi-arm block polymers were synthesized successfully and used to encapsulate the hydrophobic oxygen probe PtTFPP in their micelles. This method enabled the use of PtTFPP in the biological environment. Further, high quantum efficiency as high as 22.3% and/or long lifetime as long as 76 µs of the PtTFPP molecules in micelles under nitrogen were achieved without chemical modification of the PtTFPP. Structure influences on photophysical properties were observed among the five polymers. The results showed that the fluorine-containing micelles exhibit greater oxygen sensitivity and longer phosphorescence lifetime than the micelles formed from the fluorine-free block copolymers due to the synergy of fluorine moieties. Representative micelles **M3** formed from **P3** and PtTFPP were used for the monitoring of *E. coli* JM109 and macrophage J774A.1. cells’ respiration. Hypoxic tumor imaging of Balb/c nude mice was carried out successfully by using micelles **M3**. An obvious difference was found between the tumor and normal region because of the hypoxia condition in the tumor site. This study reported new oxygen sensors, elucidated structure influences on the photophysical properties of PtTFPP and oxygen sensing properties, and demonstrated the real-time monitoring cell respiration and in vivo hypoxia imaging at tumor sites. It is expected that this approach can be extended to other sensors and imaging probes for new multifunctional materials for further applications in clinical diagnoses and cancer therapy by taking the advantages of the materials’ biocompatibility, nontoxicity, non-invasive measurements, and long phosphorescence lifetime.

## Figures and Tables

**Table 1:** Yields, conversions and characters of the star polymers.

	Yield (%)	Conv. (%)	M_n(NMR)_	M_n(GPC)_	M_w_/M_n_	CMC (μg/mL)
4-arm-PEG-Br	79	-	10,940	3960	1.1	-
**P1a**	50	76	33,790	5340	1.3	6.9
**P1b**	44	56	45,880	7650	1.2	8.8
**P1c**	40	51	62,680	10,710	1.3	10.5
**P2**	26	34	24,780	6280	1.2	5.7
**P3**	48	49	59,220	8370	1.5	6.0

NMR = nuclear magnetic resonance spectroscopy; GPC = gel permeation chromatography.

**Table 2:** Characterization of the five kinds of PtTFPP-containing micelles.

Micelles	Diameter (nm)	^a^PDI	Con _(Polymer)_ (mg/mL)	Con _(PtTFPP)_ (mg/mL) ^b^	Encapsulation Efficiency (wt%)	Quantum Efficiency (%)	^c^*I*_0_/*I*_100_		^d^ Response Time (s)		^e^ Lifetime (μs)	
							25 °C	37 °C	t_95-r_	t_95_	Air	N_2_
**M1a**	163	0.31	0.86	0.14	42	19.5	15	25	178	29	21	73
**M1b**	142	0.27	0.38	0.13	39	22.3	15	27	204	31	22	72
**M1c**	122	0.34	0.34	0.06	18	21.5	11	28	191	30	29	70
**M2**	145	0.17	1.11	0.31	93	11.8	8	10	163	34	25	66
**M3**	126	0.15	1.90	0.30	90	16.7	14	24	198	27	18	76

^a^ Polymer dispersity index (PDI) is used to describe the molecular size distribution. ^b^ PtTFPP concentration in micelles was measured by referring PtTFPP absorption standard curve. ^c^ Phosphorescence intensity ratio under 100% N_2_ to 100% O_2_. ^d^ The values reported here are only applicable to the discussion of the system in this paper. ^e^ Micelles phosphorescence lifetime in air and N_2_ atmosphere respectively. Con: The abbreviation of concentration.

## Supplementary Materials

The following are available online at http://www.mdpi.com/1424-8220/18/11/3752/s1, Figures S1–S8: title.
