# Supplementary material for: Amphiphilic Fluorine-Containing Block Copolymers as Carriers for Hydrophobic PtTFPP for Dissolved Oxygen Sensing, Cell Respiration Monitoring and In Vivo Hypoxia Imaging with High Quantum Efficiency and Long Lifetime
Source: Sensors (Basel). 2018 Nov 2;18(11):3752. doi: 10.3390/s18113752 (PMC6263385; doi:10.3390/s18113752)
Supplement: Supplementary file 1 [file sensors-18-03752-s001.pdf]

Supporting Information

Amphiphilic fluorine-containing block copolymers as carriers for hydrophobic PtTFPP for dissolved oxygen sensing, cell respiration monitoring and *In Vivo* hypoxia imaging with high quantum efficiency and long lifetime

Jiaze Li[1,2], Yuan Qiao[2], Tingting Pan[2], Ke Zhong[2], Jiaxing Wen[2], Shanshan Wu[2,3], Fengyu Su[4,*], Yanqing Tian[2,*]

1. School of Materials Science and Engineering, Harbin Institute of Technology, Nangang District, Harbin 150001, China; 11749240@mail.sustc.edu.cn
2. Department of Materials Science and Engineering, Southern University of Science and Technology, Xili, Nanshan District, Shenzhen 518055, China; qiaoy@mail.sustc.edu.cn (Y.Q.); 11553010@mail.sustc.edu.cn (T.P.); 11612419@mail.sustc.edu.cn (K.Z.); 11510869@mail.sustc.edu.cn (J.W.); 2018090123@gdip.edu.cn (S.W.)
3. Light Chemical Technology College, Guangdong Industry Polytechnic, Haizhu District, Guangzhou 510300, China
4. SUSTech Academy for Advanced Interdisciplinary Studies, Southern University of Science and Technology, Xili, Nanshan District, Shenzhen 518055, China

* Correspondence: fysu@sustc.edu.cn (F.S.); tianyq@sustc.edu.cn (Y.T.); Tel.: +0-755-88018997

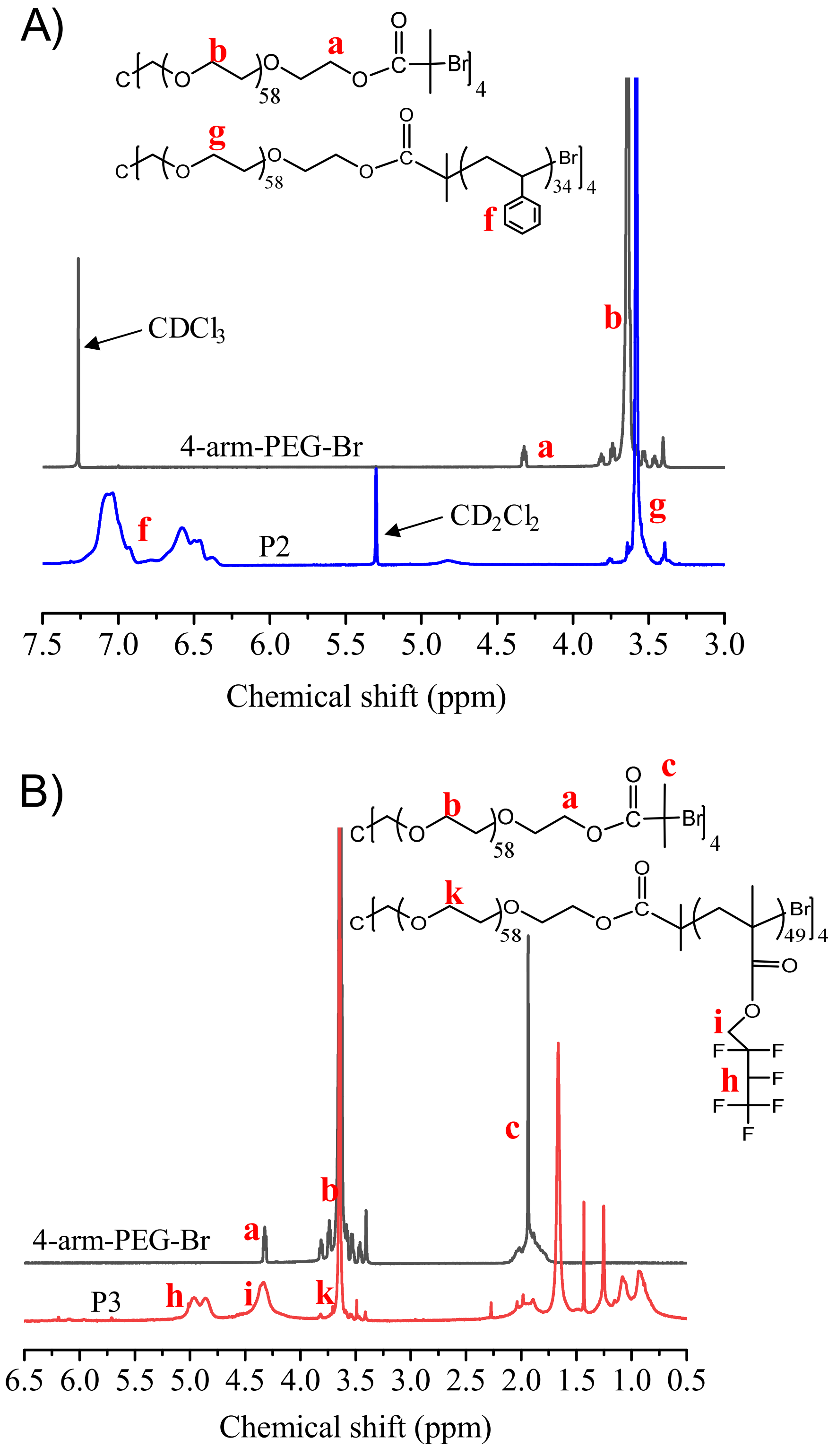

Figure S1. ^{1}H NMR spectra of **P2** (A) and **P3** (B).

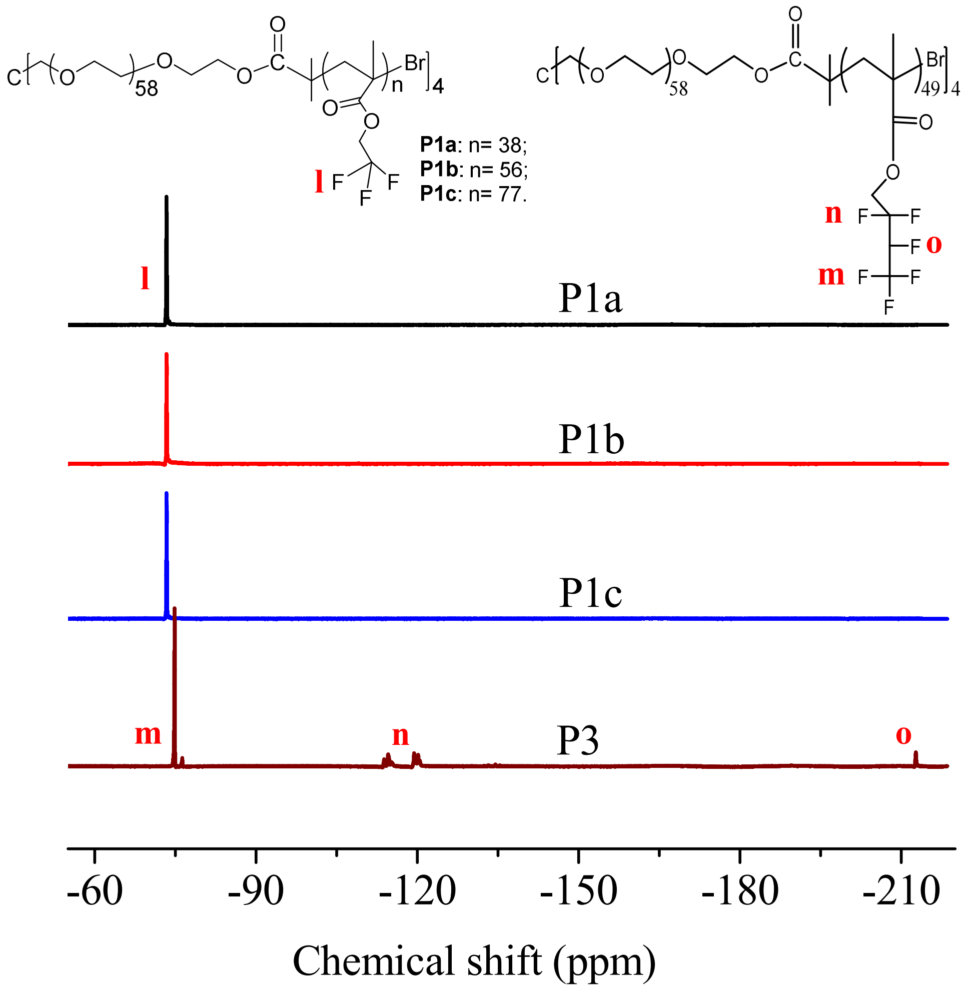

Figure S2: ^{19}F NMR spectra of **P1** series and **P3**.

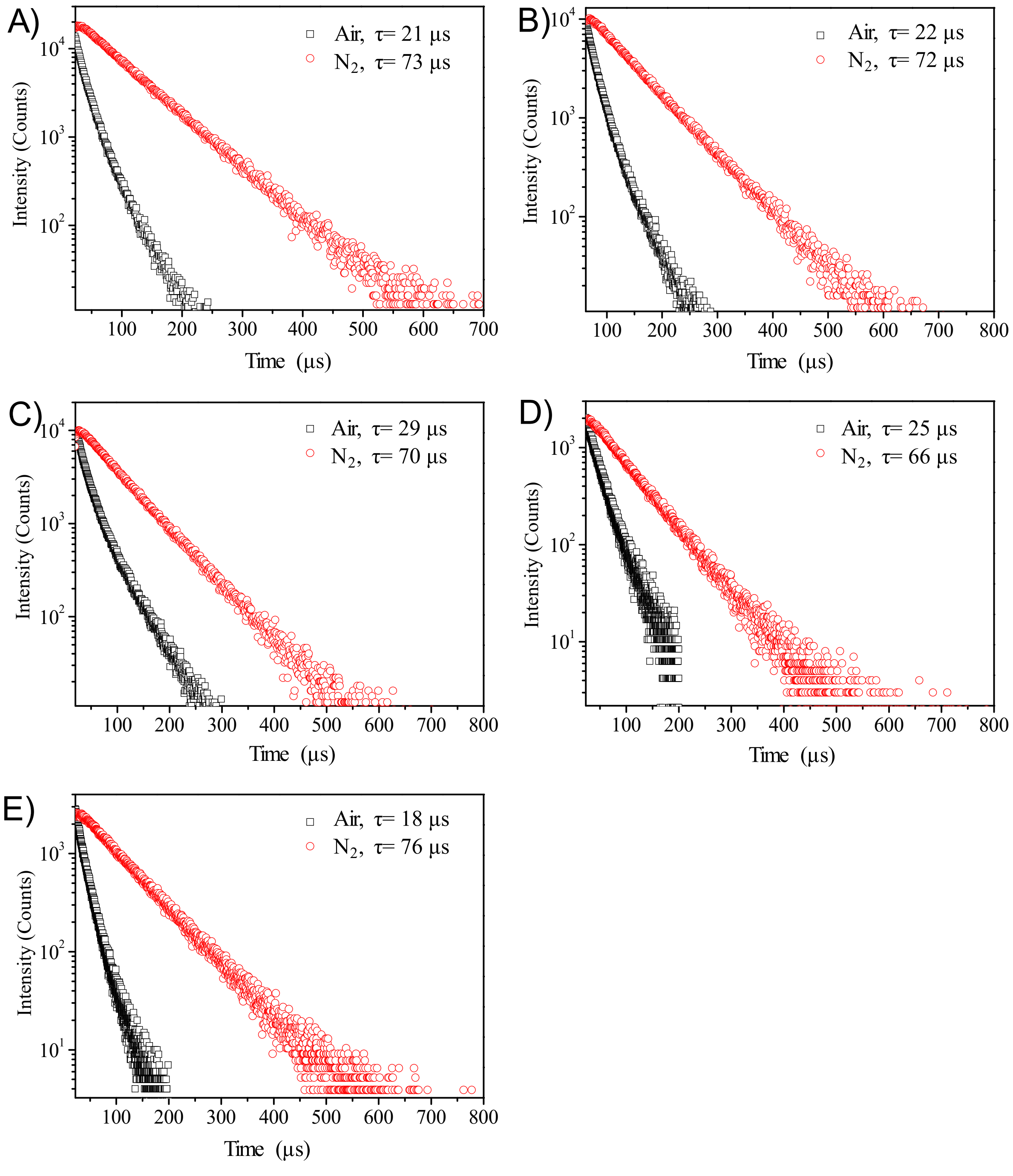

Figure S3. Phosphorescence decay curves of **M1a** (A), **M1b** (B), **M1c** (C), **M2** (D), and **M3** (E) under air and nitrogen, respectively.

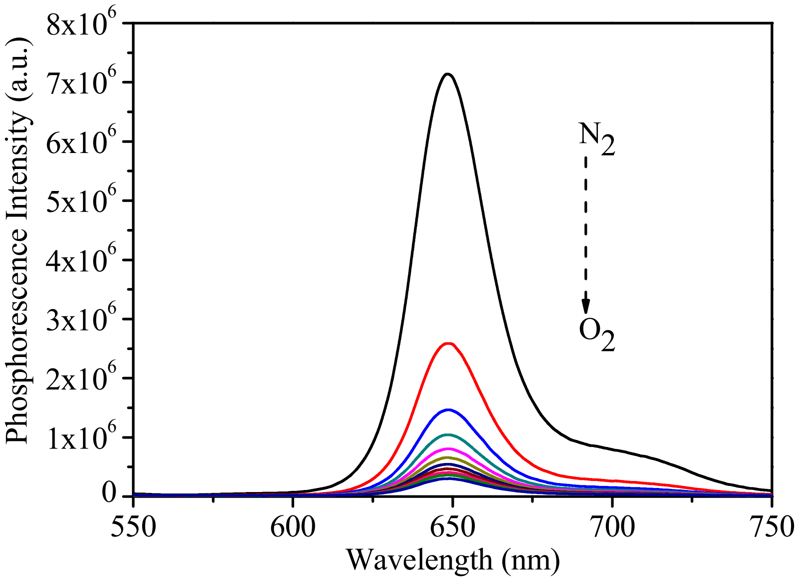

Figure S4. Oxygen responses of **M3** at 37°C.

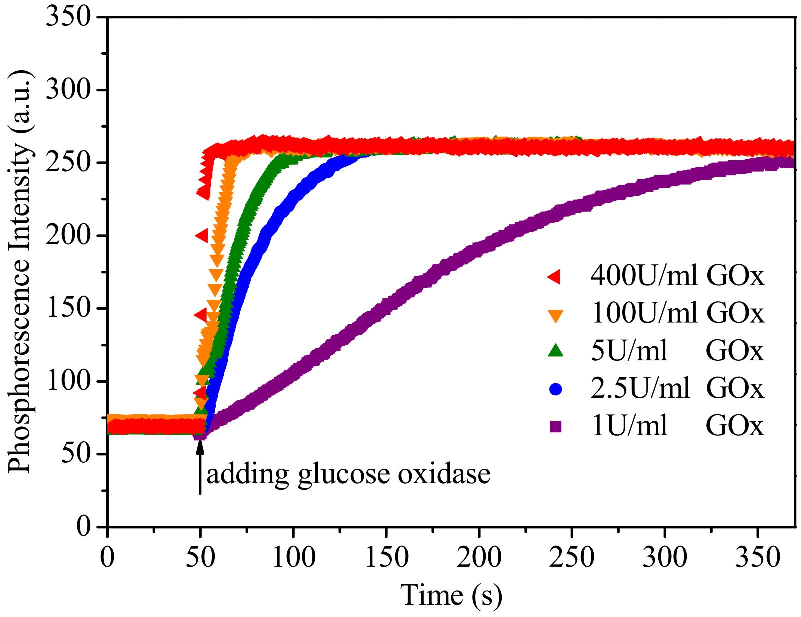

Figure S5. Phosphorescence intensity curve of **M3** in glucose solution (0.2M) when adding different concentrations of glucose oxidase for monitoring the response of **M3** to oxygen consumption.

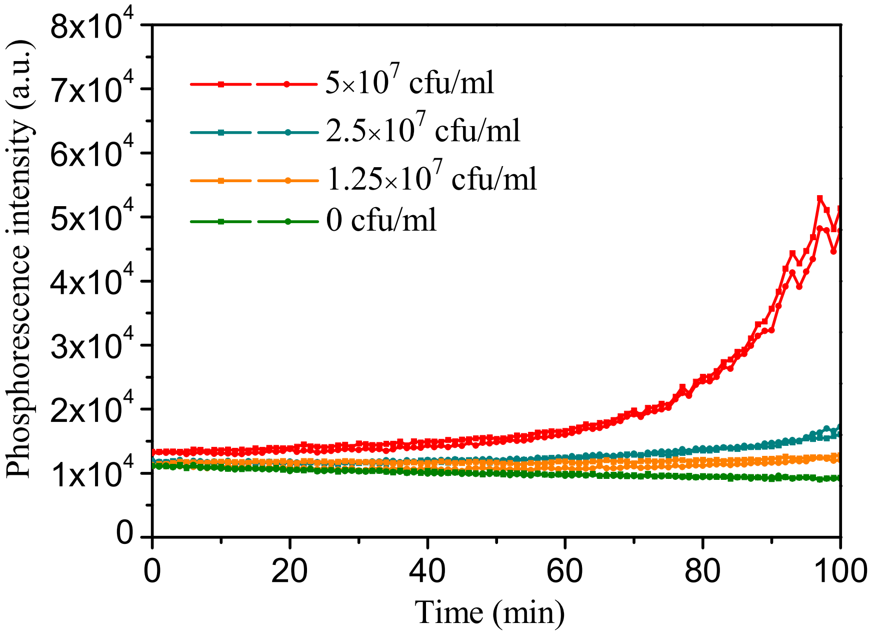

Figure S6. Dynamic phosphorescence intensity curve of **M3** measured by plate reader to determine the *E.coli* respiration without oil seal.

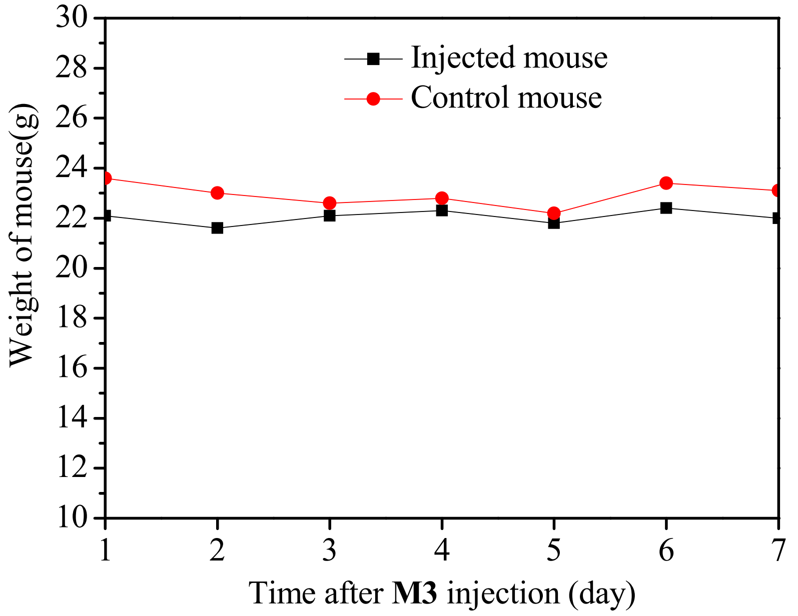

Figure S7. **M3**'s toxicity measurement of tumor-bearing mice, one mouse was intratumorally injected 50μl of **M3**, another mouse was set as control group. The experiment lasted for 7 days.

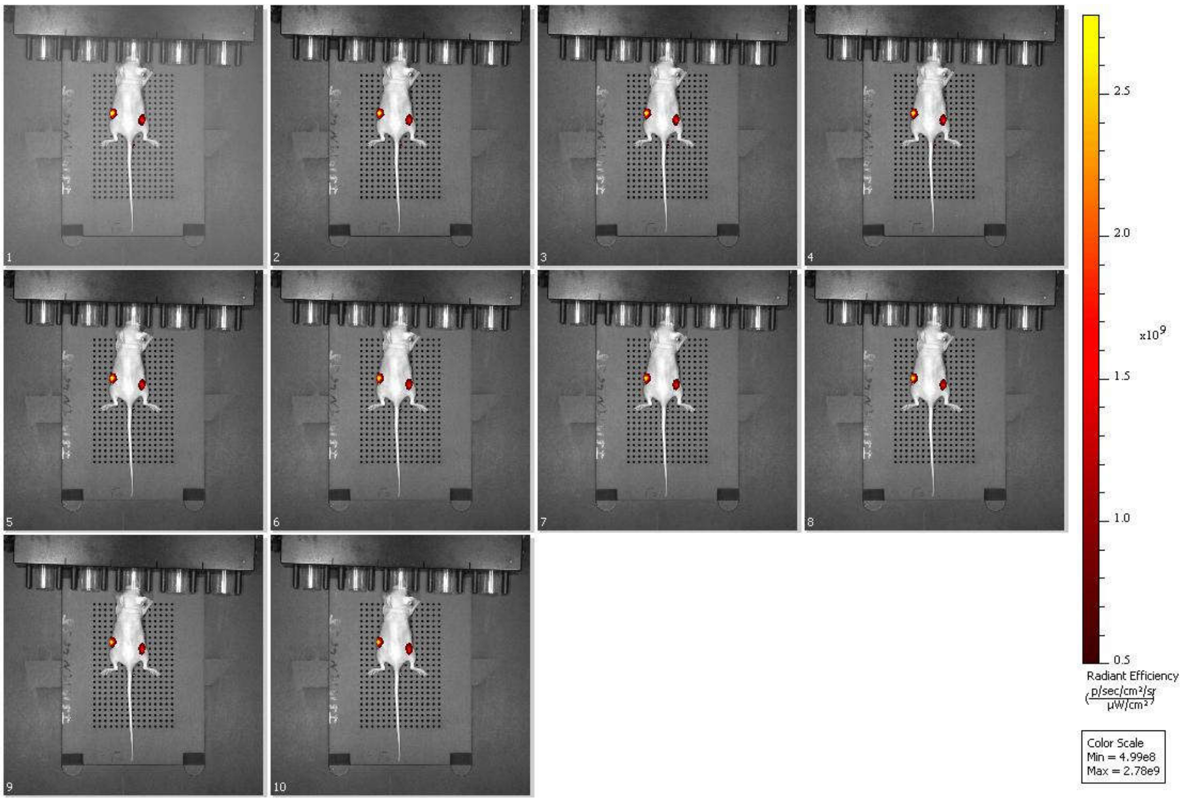

Figure S8. Phosphorescence imaging of a mouse in a time range of 10 minutes. The left side is tumor region, and the right side is normal region. After **M3** injection, the phosphorescence image was taken once per minute.
